# A Bio-inspired Hypoxia Sensor using HIF1a-Oxygen-Dependent Degradation Domain

**DOI:** 10.1038/s41598-019-43618-4

**Published:** 2019-05-08

**Authors:** Pablo Iglesias, Cristina Penas, Laura Barral-Cagiao, Elena Pazos, Jose A. Costoya

**Affiliations:** 1 Molecular Oncology Laboratory. Departamento de Fisioloxia, Facultade de Medicina and Centro de Investigación en Medicina Molecular y Enfermedades Crónicas (CiMUS). Instituto de Investigación Sanitaria de Santiago de Compostela (IDIS), Universidade de Santiago de Compostela, Santiago de Compostela, Spain; 2 Centro Singular de Investigación en Química Biolóxica e Materiais Moleculares (CiQUS), Departamento de Química Orgánica, Universidade de Santiago de Compostela, 15782 Santiago de Compostela, Spain; 3 Departamento de Química, Facultade de Ciencias and Centro de Investigacións Científicas Avanzadas (CICA), Universidade da Coruña, 15071 A Coruña, Spain

**Keywords:** Cancer, Chemical biology

## Abstract

Functional imaging has become an important tool in oncology because it not only provides information about the size and localization of the tumour, but also about the pathophysiological features of the tumoural cells. One of the characteristic features of some tumour types is that their fast growth leads to deficient intratumoral vascularization, which results in low oxygen availability. To overcome this lack of oxygen, tumoural cells activate the neoangiogenic program by upregulating the transcription factor HIF-1α. Herein we report a non-invasive *in vitro* detection method of hypoxia using designed fluorescent peptide probes based on the oxygen-dependent degradation domain of HIF-1α. The fluorescent probe retains the oxygen-sensing capability of HIF-1α, so that it is stabilized under hypoxia and readily degraded by the proteasome under normoxia, thus providing direct information of the cellular oxygen availability.

## Introduction

Some tumoural types tend to grow in a rapid and disorganized manner leading to poor intratumoral vascularization and low oxygen availability. In order to overcome this deficiency, tumour cells, just like their normal counterparts, promote the formation of neovessels by activating pathways that regulate this process of neoangiogenesis^1^. Moreover, hypoxia is a poor-prognosis microenvironmental hallmark of many solid tumours, and also influences the outcome of disseminated tumour cells contributing to their maintenance and treatment resistance^2^. In this context, the transcription factor HIF-1α stands out as the master regulator of neoangiogenesis, considering the number of genes whose expression controls when hypoxia is elicited, as well as its active role in the overall process^3^. As such, HIF-1α has been used as a prognostic marker of tumoural aggressiveness and its activity a readout of tumoural proliferation and ability to metastasize^4^.

The development of novel and more efficient techniques of visualization of tumoural processes has become a top priority in molecular imaging, particularly in functional imaging to monitor biological processes intimately related to cancer, such as metastasis and hypoxia^5^. In the last years, optical imaging methods based on fluorescence or bioluminescence have been gaining popularity in this field, thanks to the availability of a broad range of proteins and dyes^6,7^. In this context, a number of groups have recently developed several NIR (near-infrared) fluorescent proteins that enable real-time imaging free of autofluorescence interference, thus making possible to take a deep view into the tissues. Our group has previously described the design and *in vitro* and *in vivo* characterization of a genetically encoded biosensor. This sensor, which combines a fluorescent far-red protein (mCherry) and the firefly luciferase (FLuc) and is activated by the neoangiogenesis-related transcription factor HIF-1α, allowed us to differentiate tumoural masses with metastatic potential with high accuracy in a mouse model of metastasis^8^. At the same time, by fusing a fluorescent to a bioluminescent protein we obtained a bioluminescence resonance energy transfer (BRET) phenomenon, turning this fusion protein into a new class of hypoxia-sensing genetically encoded biosensor^8,9^. Recently, another genetically encoded biosensor consisting of the fluorescent protein GFP fused to the oxygen-dependent degradation (ODD) domain of the *Droshophila* homolog of HIF-1 Sima was reported^10^.

Although our biosensor demonstrated potential in hypoxia sensing, it was not directly applicable in a clinical setting because of the limitations inherently associated to biochemical sensors based on large protein constructs. Preeminent among those limitations are the need for transfection and over-expression, relatively low photostability, and large size that can lead to interference, poor biodistribution, or immune response^11,12^. In addition to avoiding those problems, peptides offer a number of advantages, including higher stability and lower immunogenicity, ease of synthesis, and the simplicity for molecular engineering, as well as better biodistribution^13^.

In this study, we take advantage of what we learned from our previous genetically encodable protein sensors, and describe a compact sensor consisting on a fluorescently-labelled peptide, corresponding to a small fraction of the ODD domain of HIF-1α, that mimics the behaviour of HIF-1α under hypoxia conditions, making possible its application for the monitorization of hypoxic activity with potential clinical applicability.

## Results and Discussion

### Biosensor design and synthesis

Hypoxia transcriptional program activation depends on hypoxia-induced stabilization of HIF-1α^14^. The molecular mechanism underlying this stabilization was a subject of great discussion due to the dispute between different models, such as the occurrence of hypothetical O_2_-binding hemoproteins or oxidases interacting with HIF-1^15^. As we now know, HIF-1α is rapidly degraded in normoxic cells upon hydroxylation of two proline residues (Pro^402^ and Pro^564^) located in its oxygen-dependent degradation domain (ODD domain)^16–18^. Upon hydroxylation, these residues are recognized by von Hippel-Lindau E3 ubiquitin ligase (pVHL), leading to poly-ubiquitination and subsequent degradation of the protein^19–21^.

**Figure 1:**
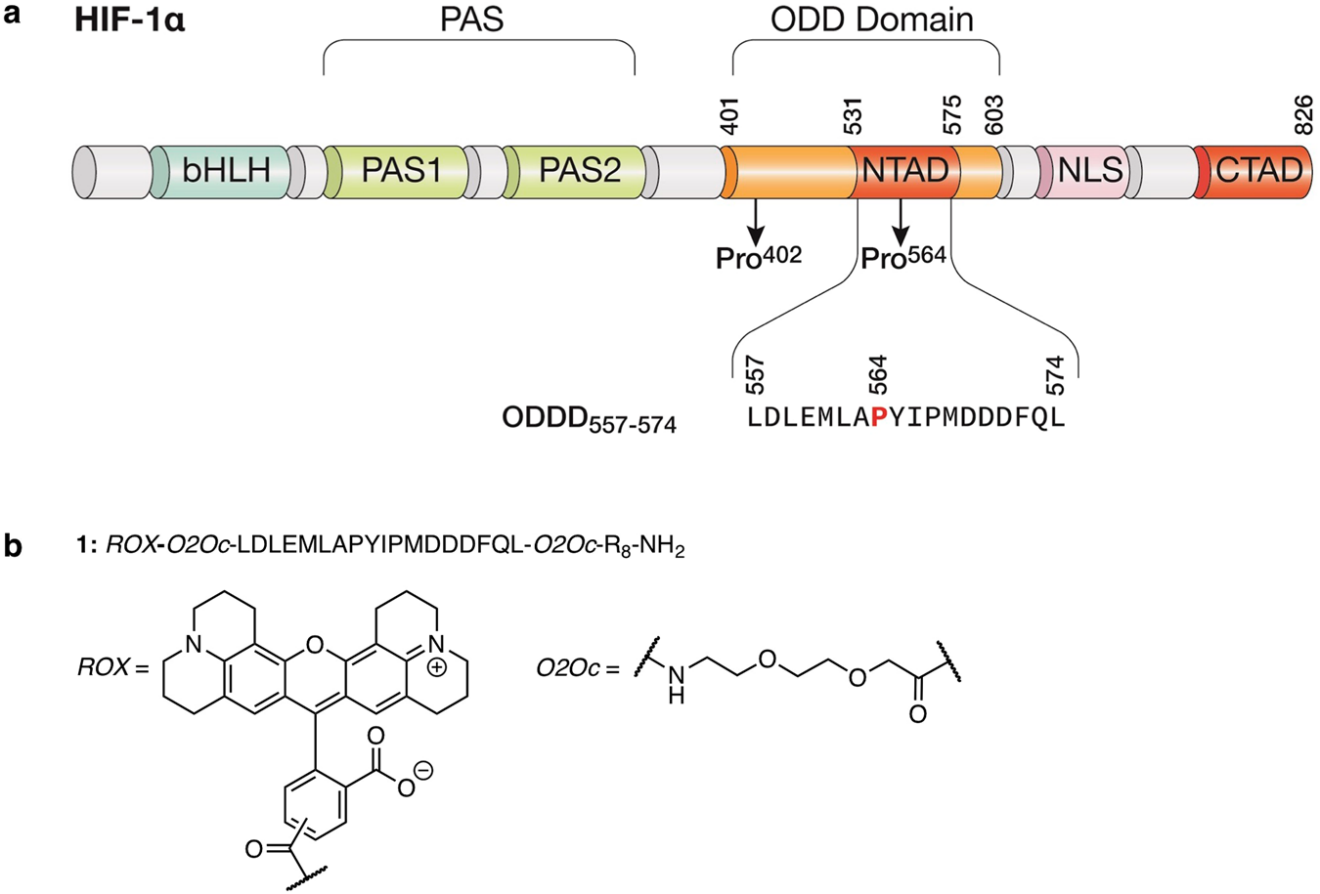
Biosensor design rationale. (**a**) Domain structures of HIF-1α. The ODD domain regulates the stability of HIF-1α via recognition by the E3 ubiquitin ligase pVHL. (**b**) Chemical structure of sensor **1**.

With the aim of developing new hypoxia tracers, we envisioned a fluorescent peptide that would mimic the effect of hypoxia on the half-life of HIF-1α. Since the total length of the HIF-1α ODD domain^16^, 203 residues as depicted in Fig. 1a, makes unpractical its chemical synthesis and the incorporation of the sensing unit, we decided to use a short 16-mer peptide derived from the HIF-1α ODD domain, Leu^557^ to Leu^574^. This peptide has been previously reported to be profusely hydroxylated in the Pro^564^ residue during normoxia, leading to pVHL-mediated degradation^22^, and thus retains the oxygen-sensing properties of HIF-1α^23^. As a general feature, the new sensor has three small modules with different functions: an octa-arginine peptide that mediates cell internalization^24,25^, a central domain from the HIF-1α degradome able to sense low oxygen levels (**1**: ^557^LDLEMLAPYIPMDDDFQL^574^)^26^ and the 5,(6)-ROX fluorochrome, a long-wavelength rhodamine characterized by a similar emission profile to mCherry^27,28^, good stability^29,30^ and high quantum yield (0.92)^30^, that acts as fluorescent reporter of the integrity of the peptide for *in vitro* and *in vivo* imaging (Figs 1 and S1). These three modules are connected by short PEG linkers (O2Oc)^31^ to avoid interference in the recognition of the HIF central domain by prolyl hydroxylase.

In contrast with other fluorescent sensors of proteins, such as those based on solvatochromic dyes that increase their emission intensity in hydrophobic environments, i.e. protein pockets^32^, or those based in energy transfer processes^33^, in this case the sensing mechanism relies on the higher lifetime of the ODD domain sequence under hypoxic conditions than under normoxia. Since the degradation of HIF-1α is triggered by hydroxylation of the Pro^564^ in the ODD domain, we expected that the proteolytic stability of a fluorescent peptide containing the appropriate region of ODD domain would be under the same control as HIF-1α, so that the accumulated fluorescence would reflect the changes in oxygen availability in the different tissues.

Peptide **1** was synthesized following standard Fmoc solid phase peptide synthesis (SPPS) protocols, and as a final step labelled at their deprotected *N-*terminus with 5(6)-ROX. Once purified by reversed-phase HPLC and lyophilized, peptide **1** was dissolved in PBS and its excitation and emission spectra were recorded showing the characteristic fluorescence profile of ROX fluorophore conjugates (Fig. S4, excitation 585 nm, emission 608 nm)^30^.

### Internalization and stabilization of sensors by hypoxia-mimetic agents

**Figure 2:**
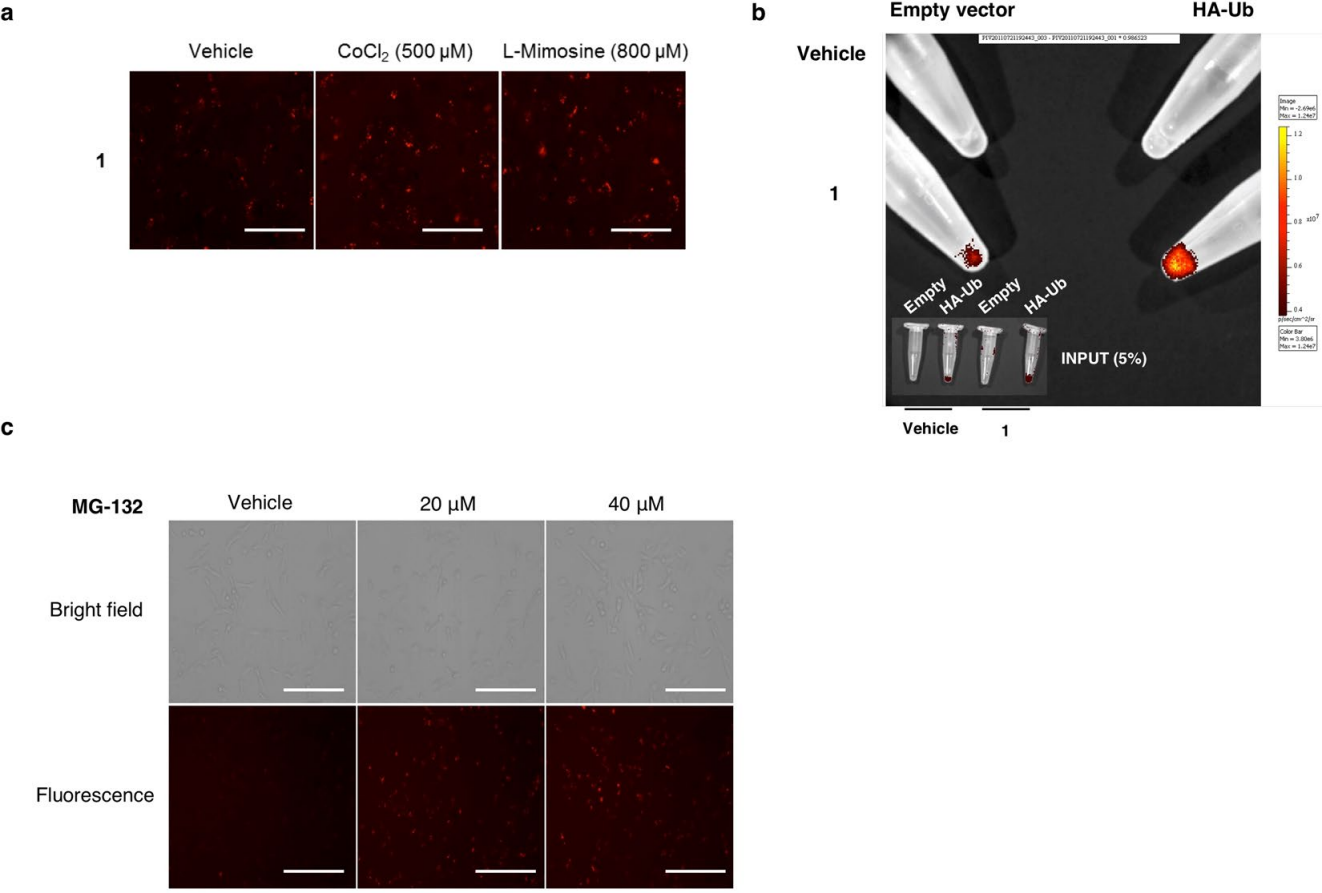
(**a**) Hypoxia mimetics inhibit sensor degradation. Cells were incubated with hypoxia sensor **1** in the presence of CoCl_2_ (500 μM) and L-Mimosine (800 μM). Scale = 50 μm. (**b**) Ubiquitination of hypoxia sensor **1** is demonstrated by immunoprecipitation of HA-tagged ubiquitinated substrate. (**c**) Proteasome degrades sensor **1**. Proteasome inhibitor MG132 inhibits in a dose response manner the degradation of hypoxia probe **1** in MDA-MB 231 cells. Scale = 100 μm.

Human breast cancer cells MDA-MB 231 were treated with **1**. Cells were incubated with vehicle or the hypoxia-mimetic agents: CoCl_2_, an inhibitor of the interaction between HIF-1α and the ubiquitin ligase von Hippel-Lindau protein (pVHL)^34^, and the prolyl hydroxylase inhibitor l-Mimosine^35^. As can be observed in Fig. 2a, the fluorescent peptide **1** is stabilized in the presence of both molecules that mimic hypoxia, thus demonstrating our hypothesis for the design of the probe based on an inhibition of the sensor degradation in a HIF-dependent hypoxic response. Although, the octa-arginine cell-penetrating peptide has been previously described as a potent inhibitor of proteasome activation^36^, this new fluorescent hypoxic sensor displays an improved stabilization when the level of signal in vehicle-treated cell is compared with CoCl_2_ and l-Mimosine-treated cells.

### Hypoxia targets sensors specifically via ubiquitin-proteasome degradation

One of our main concerns was the way this peptidic probe would be processed within cells. Ideally, the probe would be eliminated in the same manner as HIF-1α, i.e., degraded by the proteasome upon hydroxylation of the ODD domain. To further investigate into this issue, we transfected cells with a plasmid encoding anti-hemagglutinin (HA)-tagged ubiquitin, in order to perform an immunoprecipitation, and treated these cells with probe **1**. In cells transfected with HA-tagged ubiquitin the enrichment of hypoxia sensors by (HA) immunoprecipitation shows that the degradation of the sensor is ubiquitin proteasome-regulated (Fig. 2b). Moreover, if cells incubated with hypoxic sensor **1** are treated with a proteasome inhibitor (MG-132), a dose-dependent effect on proteasome degradation of fluorescent signal is demonstrated (Fig. 2c).

**Figure 3:**
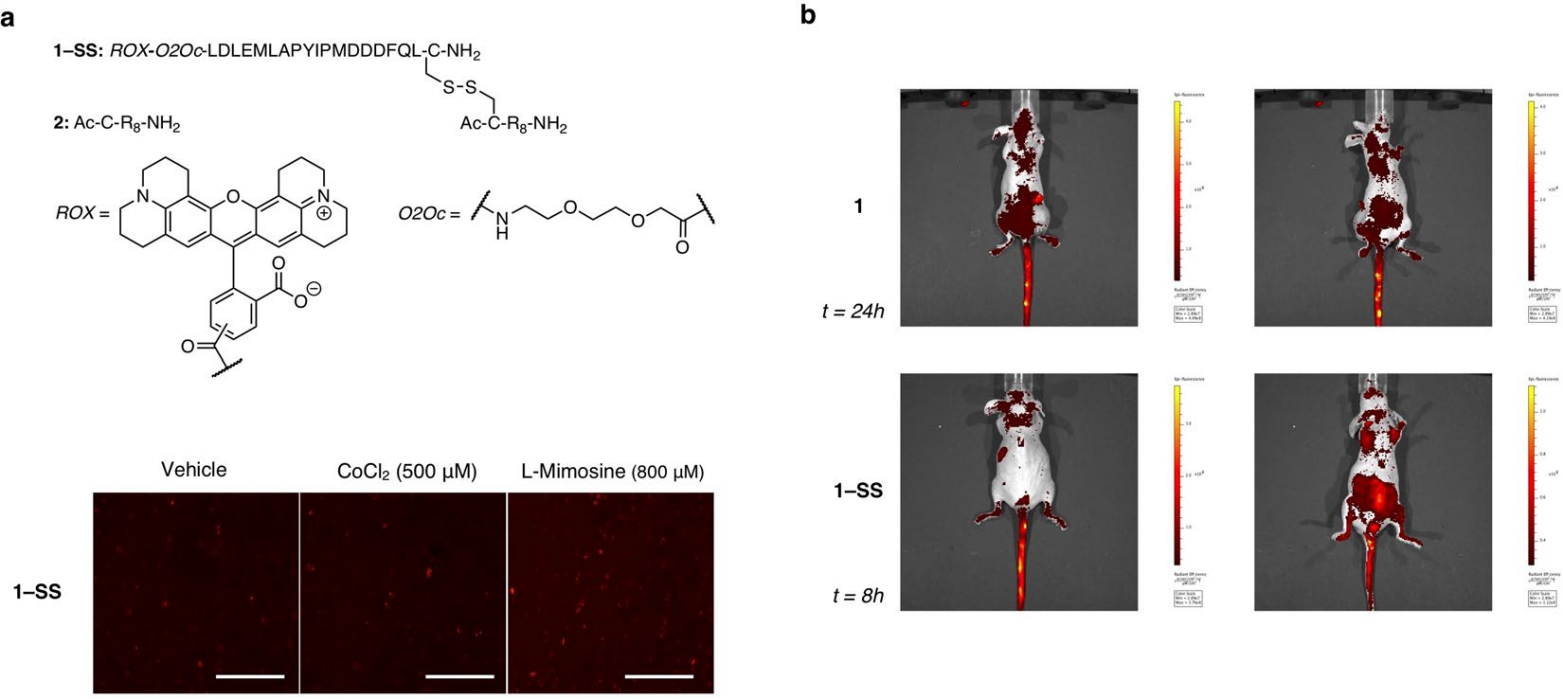
(**a**) (upper pannel) Chemical structures of peptide **2** and probe **1-SS**. (lower panel) Hypoxia mimetics also inhibit sensor **1-SS** degradation. Cells were incubated with hypoxia sensor **1-SS** in the presence of CoCl_2_ (500 μM) and L-Mimosine (800 μM). Scale = 50 μm. (**b**) Comparation between the clearance time of sensor **1** (up) and **1-SS** (down) *in vivo*. Images in prone (left) and supine position (right) were taken every two hours during the first eight hours upon sensor administration and one final time at 24 hours post-injection. Images shown correspond to the last time point where specific signal from the sensor was detected.

On the other hand, depending on their cargo, octa-arginines are also known to induce nuclear uptake^37,38^, thus probably extending the half-life of the probe within the cell and delaying its clearance. Given that disulfide bridges are sensitive to the reductive environment of the cytoplasm, we decided to synthesize a new probe, **1–SS**, in which the octa-arginine peptide and the ODDD-based sensing unit are connected through a disulfide bond, with the purpose of reducing its half-life (Fig. 3a upper panel). The cleavage of the disulfide bond in the cytosol once the compound is internalized^39^ should give the corresponding octa-arginine and ODD domain fragments, thus facilitating the degradation of the sensing unit.

The strategy followed for the synthesis of the *N-*terminus ROX-conjugated peptide **1–SS** is described in detail in the methods section and supporting information (Fig. S1). In short, the precursor peptide **1-Cys**, that contains the previously described ODDD sequence with an additional cysteine residue, as well as peptide **2**, featuring the octa-arginine sequence with an appended cysteine residue, were synthesized following standard solid phase protocols, and HPLC-purified. Peptide **2** was then activated with 5,5-dithio-bis-(2-nitrobenzoic acid) (Ellman’s reagent) and incubated with **1-Cys** to promote a disulfide exchange reaction, that provided the desired conjugate **1-SS**. Probe **1-SS** showed the same excitation and emission profile than peptide **1** (Fig. S5).

As was expected, probe **1–SS** showed increased rate of degradation, both *in vitro* and *in vivo*. Treated MDA-MB-231 cells with hypoxia sensors in the presence of CoCl_2_ and l-Mimosine revealed a faster reduction of the fluorescence intensity (Fig. 3a lower panel). Along with faster clearance rates we also observed a reduction in background fluorescence and an enhanced sensitivity with increasing concentrations of CoCl_2_ when compared to probe **1** (Fig. S6). Indeed, this reduction was also observed when probe **1** and **1–SS** were administered *in vivo* to mice. As can be observed in Fig. 3b, 1**–SS** showed a lower signal at 8 h post administration than **1** at 24 h. The fast renal elimination of fluorophore results in strong bladder fluorescence when **1–SS** was administered. Additional time points can be found in Fig. S7.

In summary, we have shown that the stabilization effect of hypoxia on the half-life of HIF-1α can be exploited for the design of fluorescent sensors of hypoxia. We have therefore designed and tested a small synthetic peptide biosensor, based on the ODD domain of HIF-1α and modified with an octa-arginine cell-penetrating peptide and a fluorescent dye, whose proteolytic stability is controlled under hypoxia and the fluorescence signal depends on the oxygen availability. We have further confirmed that the probe is degraded by the proteasome upon hydroxylation of the ODD domain. Moreover, the reengineering of the initial peptide probe, by means of adding a disulfide bridge between the octa-arginine vector and the ODDD sensing unit, accelerates its degradation both *in vitro* and *in vivo*. Although other approaches have been developed around a similar concept using genetically encoded biosensors^10,40^ or nitroaromatic compounds^41,42^, our probe combines excellent cell internalization with faster clearance rates which improves its sensitivity and applicability in monitoring hypoxic environments. Therefore, these tracers could serve not only to pinpoint tumoural masses, but also to inform us about their metastatic potential and aggressiveness and, at the same time, gain a deeper view on the tumour microenvironment that modern imaging techniques do not provide.

## Materials and Methods

### Chemicals and instrumentation

All peptide synthesis reagents and amino acid derivatives were purchased from GL Biochem (Shanghai) and *NovaBiochem*. Amino acids were purchased as standard Fmoc protected amino acids: Fmoc–Leu–OH, Fmoc–Asp(OtBu)–OH, Fmoc–Glu(OtBu)–OH, Fmoc–Met–OH, Fmoc–Ala–OH, Fmoc–Pro–OH, Fmoc–Tyr(tBu)–OH, Fmoc–Ile–OH, Fmoc–Phe–OH, Fmoc–Gln(Trt)–OH, Fmoc–Arg(Pbf)–OH, Fmoc–Ser(tBu)–OH, Fmoc–Thr(tBu)–OH, and Fmoc–Cys(Trt)–OH. Fmoc–O2Oc–OH was purchased from *Iris Biotech GMBH* (Cat. #: FAA1435), and 5(6)-ROX, SE was purchased from *Invitrogen* (Cat. #: C1309). *C*-terminal amide peptides were synthesized on a 0.025 mmol scale using a 0.23 mmol/g loading Fmoc-PAL-PEG-PS resin from *Applied Biosystems*. All other chemicals were purchased from *Sigma-Aldrich*. All solvents were dry and synthesis grade, except DMF for peptide synthesis. Peptides were synthesized using a PS3 automatic peptide synthesizer from *Protein Tecnologies*.

High-Performance Liquid Chromatography (HPLC) was carried out with an *Agilent* 1100 *series* Liquid Chromatograph Mass Spectrometer system. Analytical HPLC was run using a Zorbax Eclipse XDB-C_8_ (5 µm) 4.6 × 150 mm analytical column (*Agilent*). Purification of the peptides was performed on a Jupiter Proteo 90 A (4 µm) 10 × 250 mm reversed-phase column (*Phenomenex*). The usual gradient used for analytical and semi-preparative HPLC was 15 → 95% CH_3_CN, 0.1% TFA/H_2_O, 0.1% TFA over 30 min. Electrospray Ionization Mass Spectrometry (ESI MS) was performed with an *Agilent* 1100 Series LC/MSD VL G1956A model in positive scan mode by direct injection of the purified peptide solution. Matrix-assisted laser desorption ionization time-of-flight mass spectrometry (MALDI-TOF MS) was performed with a *Bruker* ultraflex III TOF/TOF, using sinapinic acid as matrix.

ROX-modified peptides appear as overlapping pair of peaks in the HPLC traces due to the use of the commercial a mixture of 5-(and 6)-Carboxy-X-Rhodamine isomers in the synthesis.

### Peptide synthesis

The amino acids were coupled in 4-fold excess using 2-[(1*H*-benzotriazol-1-*yl*)-1,1,3,3-tetramethyluronium hexafluorophosphate (HBTU) as coupling agent. Each amino acid was activated for 30 s in DMF before being added onto the resin. Peptide couplings were conducted for 30 to 45 min. The temporal Fmoc protecting group was removed by treating the resin with 20% 4-methylpiperidine in DMF solution for 10 min.

#### 5(6)-ROX, Succinimidyl Ester (SE) coupling

The *N-*terminus-deprotected peptides **1** and **1-Cys**, attached to the resins (0.025 mmol), 0.195 M *N*,*N-*diisopropylethylamine (DIEA) in DMF (1 mL, 0.195 mmol) and 5(6)-ROX, SE (12.5 mg, 0.02 mmol) were mixed and the resin suspension was shaken for 4 h. After filtration, the resin was washed with DMF (3 × 1 mL × 3 min) and CH_2_Cl_2_ (3 × 1 mL × 3 min) and dried under argon.

#### Peptide 2 N-terminus-acetylation

After the final Fmoc deprotection step using standard conditions (20% 4-methylpiperidine/DMF), peptide **2** (0.025 mmol) was acetylated by treatment with 20% Ac_2_O in DMF (2.5 mL) and 0.195 M DIEA in DMF (1.75 mL) for 45 min. After filtration, the resin was washed with DMF (3 × 5 mL × 3 min) and CH_2_Cl_2_ (3 × 5 mL × 3 min) and dried under a current of argon.

#### Cleavage and deprotection of semipermanent protecting groups

The resin-bound peptides **1**, **1-Cys**, and **2** (0.025 mmol), were placed in a 50 mL tube to which 10 mL of the cleavage cocktail (2% anisole, 3% ethanedithiol (EDT), 5% thioanisole and 90% TFA) were added. These mixtures were shaken for 3.5 h. The resins were then filtered, and the TFA filtrate was concentrated to a volume of approximately 1 mL. The residue was added to ice-cold diethyl ether (20 mL). After 10 min, the precipitate was centrifuged and washed again with 10 mL of ice-cold ether and centrifuged. The solid residue was dried under argon and redissolved in acetonitrile/water 1:1 (1 mL) and purified by semi-preparative reversed-phase HPLC. The collected fractions were lyophilized and stored at −20 °C.

#### Peptide 2 activation with Ellman’s reagent

Peptide **2** (10 mg, 0.007 mmol) was dissolved in 10 mM Tris·HCl, pH 7.5, 100 mM NaCl (0.65 mL). 5,5-dithio-bis-(2-nitrobenzoic acid) (5.6 mg, 0.014 mmol) was dissolved in a separate vial in 0.35 mL of 10 mM Tris·HCl, pH 7.5, 100 mM NaCl/MeCN (3:2). Both solutions were mixed, and the resulting mixture was shaken for 2 h. Then, the crude was purified by semi-preparative reversed-phase HPLC, and the collected fractions were lyophilized and stored at −20 °C.

#### Coupling of peptide 1-Cys with Ellman’s-conjugated peptide 2

Ellman’s-conjugated peptide **2** (5.8 mg, 0.0036 mmol) was dissolved in 10 mM Tris·HCl, pH 7.5, 100 mM NaCl (0.6 mL), and 0.4 mL of a peptide **1-Cys** (5 mg, 0.0017 mmol) solution in 10 mM Tris·HCl, pH 7.5, 100 mM NaCl/MeCN (1:1), were added. The resulting mixture was shaken for 2 h, then the crude was purified by semi-preparative reversed-phase HPLC, and the collected fractions were lyophilized and stored at −20 °C.

### Fluorescence spectroscopy

Fluorescence spectra were recorded with a Jobin*−*Yvon *Fluoromax−3* (*FluorEssence*™), coupled to a *Wavelength Electronics* LFI−3751 temperature controller. All measurements were made with the following settings: increment: 1.0 nm; integration time: 0.50 s; excitation slit width: 2.0 nm; emission slit width: 2.0 nm; excitation wavelength 585 nm. The emission spectra were recorded from 595 to 700 nm. Peptide stock solutions were quantified spectroscopically taking a molar extinction coefficient of 78000 M^−1^ cm^−1^ at 579 nm^43^.

### Cell culture and treatments

Human breast cancer cells MDA-MB 231 were maintained at 37 °C and 5% CO_2_ in RPMI-1640 (*Sigma–Aldrich*), supplemented with 10% fetal bovine serum (*Fisher Scientific*). Cells were seeded at a density of 2·10^5^ cells per well in 6-multiwell plates 24 h prior treatment. For hypoxia induction, they were treated with either CoCl_2_ (*Sigma–Aldrich*) at a final concentration of 500 μM, or L-Mimosine (*Sigma–Aldrich*) at a final concentration of 800 μM. Treatment with each peptidic sensor (500 ng/mL) was carried out in the presence of the aforementioned inhibitors. After three hours of incubation, supernatants containing the probes and either CoCl_2_ or L-Mimosine were aspirated, cells were washed once with PBS 1X and fresh medium, containing again either CoCl_2_ or L-Mimosine, was added to the cells for an extended incubation of three hours. Treatment with proteasome inhibitor MG-132 (*Sigma-Aldrich*) was carried out for 6 hours at final concentrations of either 20 μM or 40 μM, upon pre-treatment of the cells with the probes, as described previously. Fluorescent probes internalization was quantified using ImageJ (*NIH*). Quantified fluorescence was expressed as values of *corrected total cell fluorescence* (CTCF), which was calculated using the formula CTCF = integrated density – (area of selected cell × mean fluorescence of background readings)^44^.

### Immunoprecipitation

MDA-MB 231 cells were seeded at a density of 2·10^5^ cells per well in 6-multiwell plates twenty-four hours prior transfection. Each well was transfected with 2 μg of either pcDNA3-Ub-HA or empty vector pcDNA3 (*Invitrogen*) using Superfect (*Qiagen*) as transfection reagent. Twenty-four hours upon transfection culture medium was removed, cells were washed carefully with phosphate buffered saline (PBS; *Sigma-Aldrich*) and fresh culture medium containing the peptidic sensor **1** was added onto the cells. PBS was used as mock treatment. Five hours upon treatment with the sensor, cells were lysed in native immunoprecipitation buffer, 20 mM Tris·HCl pH 8, 137 mM NaCl,10% glycerol,1% Nonidet P-40 (NP-40), 2 mM EDTA, containing the protease inhibitors sodium orthovanadate (1 mM), PMSF (2 mM) and aprotinin (1 μg/mL) (*Sigma-Aldrich*). Each immunoprecipitation contained 600 μg of whole protein extract and were incubated overnight with 5 μg of anti-hemagglutinin HA.11 (Clone 16B12) antibody (*Abcam*) at 4 °C. Equal amounts of rabbit immunoglobulins (*Sigma-Aldrich*) were used as control. HA-tagged ubiquitins were immunoprecipitated using Protein G-coupled agarose (*Santa Cruz*), subsequently washed with immunoprecipitation buffer and eluted under native conditions. Fluorescence images were taken using an IVIS Spectrum system (*Perkin-Elmer*).

### Animal studies

Two female BALB/c nude mice (*Janvier*) of twelve weeks of age were injected in the tail-vein with 10 mg/Kg of either **1** or **1–SS** prior to commence the experiment. Fluorescence pictures from injected mice and a non-treated mouse used as a negative control were taken every two hours using the IVIS system during the first 8 hours, and then one final time at 48 hours post-injection. Specific fluorescence images were acquired using the 570 nm and 620 nm filter for excitation and emission, respectively, while for background fluorescence the 465 and 620 nm were used. Substraction of background fluorescence was performed with the IVIS system software following the guidelines set out by the manufacturer. Mice were housed in specific pathogen-free (SPF) conditions, following FELASA (Federation of European Laboratory Animal Science Associations) and institutional guidelines. All animal procedures were approved and performed according to the guidelines set out by the Institutional Ethics Committee for Animal Experimentation of the Universidade de Santiago de Compostela (Protocol No 15005AE/07/FUN01/FIS02/JACP1).

## Supplementary information

Supplementary Information

## Data Availability

All data are available from the corresponding authors on reasonable request.
