# Supplementary material for: A Bio-inspired Hypoxia Sensor using HIF1a-Oxygen-Dependent Degradation Domain
Source: Sci Rep. 2019 May 8;9:7117. doi: 10.1038/s41598-019-43618-4 (PMC6506541; doi:10.1038/s41598-019-43618-4)
Supplement: Supplementary file 1 — Supplementary Information [file 41598_2019_43618_MOESM1_ESM.pdf]

SUPLEMENTARY INFORMATION

A Bio-inspired Hypoxia Sensor using HIF1a-Oxygen-Dependent Degradation Domain

Pablo Iglesias[1], Cristina Penas[2], Laura Barral-Cagiao[1], Elena Pazos[3*], Jose A. Costoya[1*]

1. Molecular Oncology Laboratory. Departamento de Fisioloxia, Facultade de Medicina and Centro de Investigación en Medicina Molecular y Enfermedades Crónicas (CiMUS). Instituto de Investigación Sanitaria de Santiago de Compostela (IDIS). Universidade de Santiago de Compostela, Santiago de Compostela, Spain.

2. Centro Singular de Investigación en Química Biolóxica e Materiais Moleculares (CiQUS), Departamento de Química Orgánica, Universidade de Santiago de Compostela. 15782 Santiago de Compostela, Spain.

3. Departamento de Química, Facultade de Ciencias and Centro de Investigacións Científicas Avanzadas (CICA), Universidade da Coruña, 15071 A Coruña, Spain.

** Corresponding authors*

E-mail: elena.pazos@udc.es; josea.costoya@usc.es

Outline of the peptide synthesis and characterization

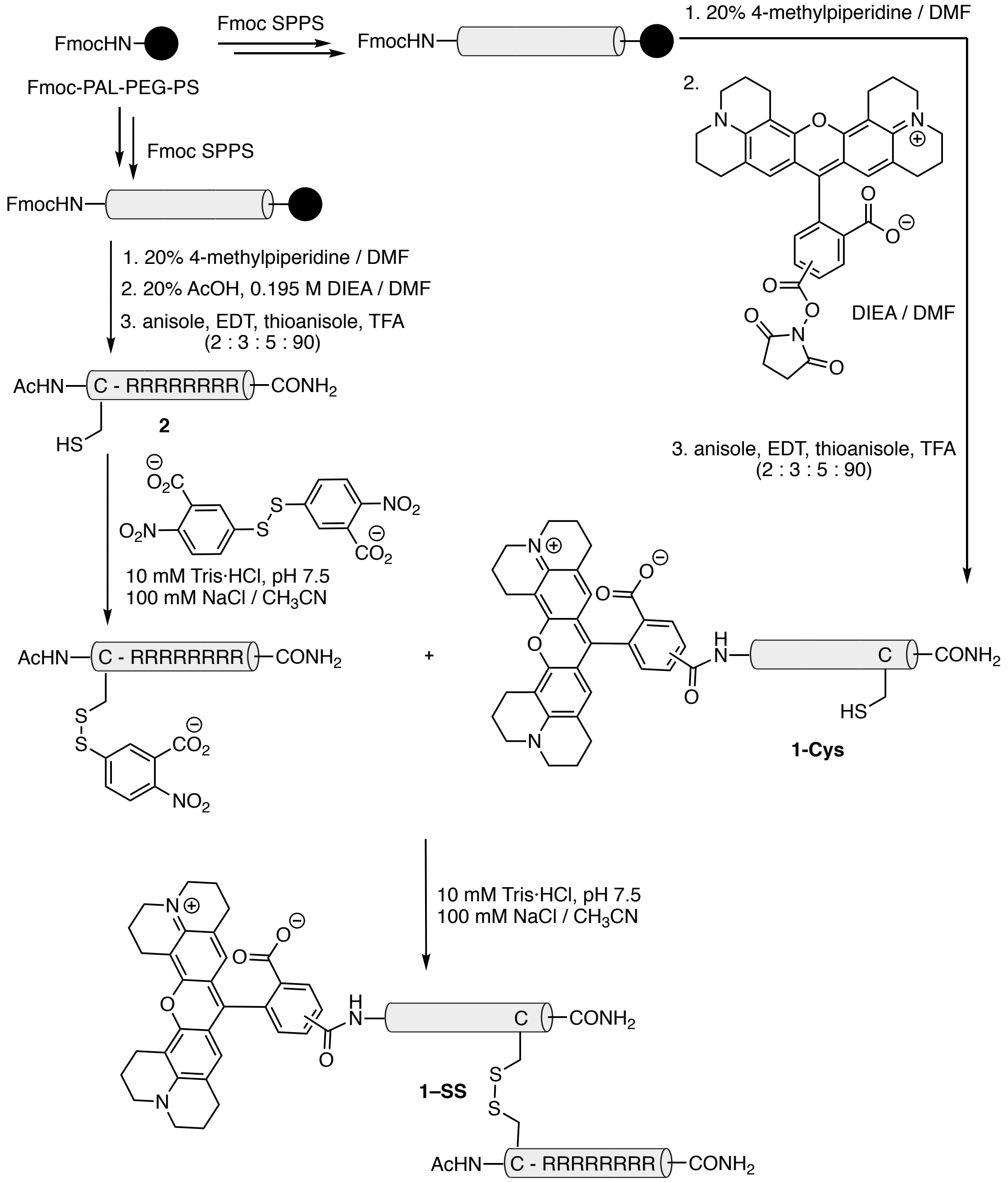

Figure S1. Synthesis of peptide **1–SS.**

1: MALDI-TOF MS: $[MH]^+$ calcd for $C_{190}H_{294}N_{56}O_{48}S_2$ = 4193,1800, found = 4193.5331. (8.7 mg, 8% yield for 0.025 mmol scale).

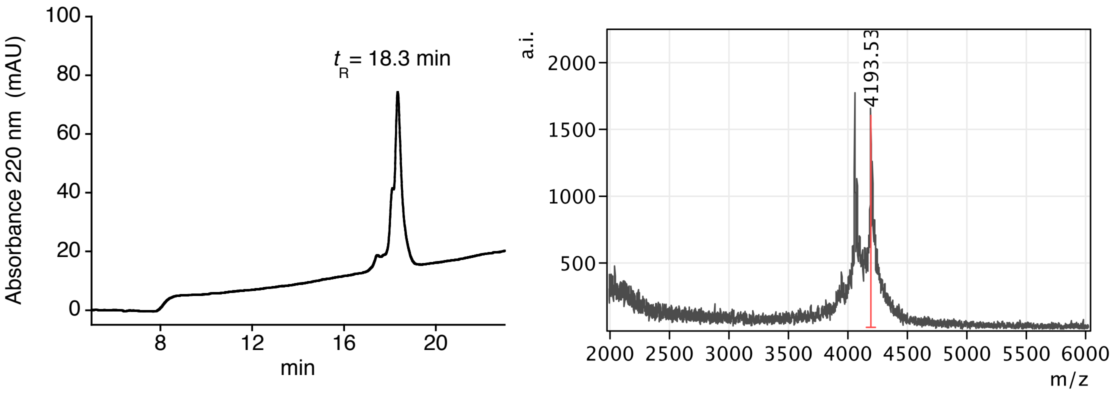

Figure S2. Left: HPLC trace, and **Right:** MALDI-MS spectrum of peptide **1.**

1–SS: MALDI-TOF MS: $[MH]^+$ calcd for $C_{192}H_{296}N_{58}O_{48}S_4$ = 4311,1460, found = 4314.7993.
(1.8 mg, 24% yield for 0.0017 mmol scale).

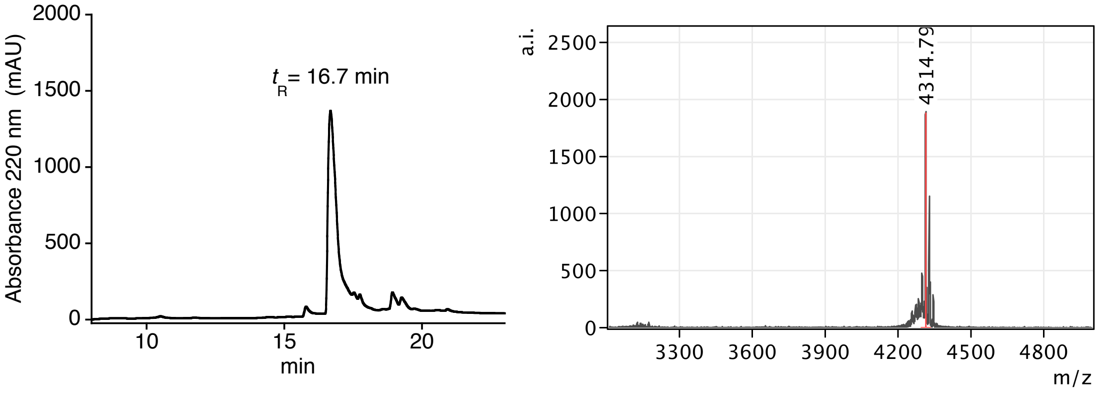

Figure S3. Left: HPLC trace, and **Right:** MALDI-MS spectrum of peptide **1–SS.**

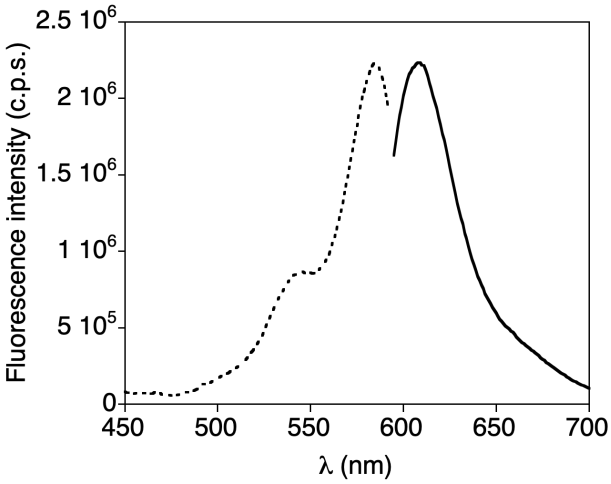

Figure S4: Excitation (dotted line) and emission (solid line) of a 1μM solution of peptide **1** in PBS**.**

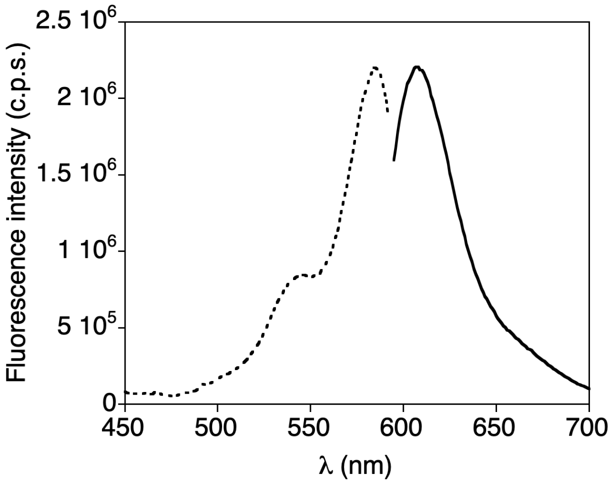

Figure S5: Excitation (dotted line) and emission (solid line) of a 1μM solution of peptide **1–SS** in PBS**.**

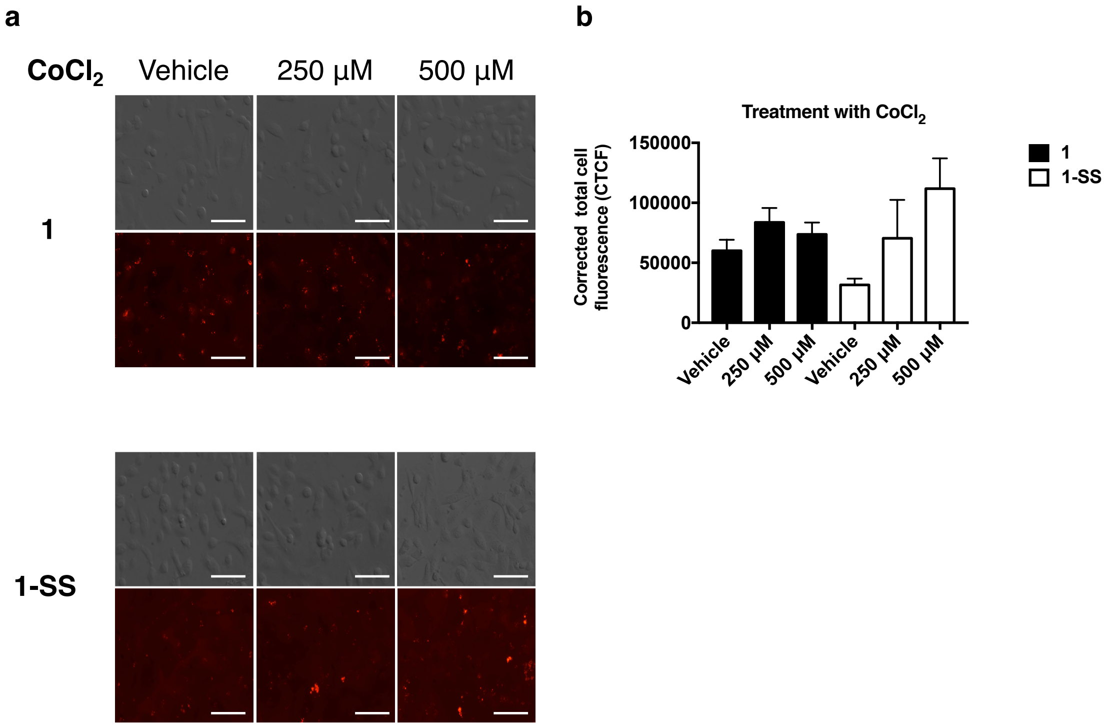

Figure S6: (**a**) Micrographs of MDA-MB-231 treated with the peptidic sensors **1** and **1–SS** (500 ng/mL). Cells were treated with $CoCl_2$ as well at the indicated concentrations. Images were taken 3 hours upon treatment at 40X magnification. Scale = 50 µm. (**b**) Quantification of the corrected total cell fluorescence (CTCF), error bars represent SEM.

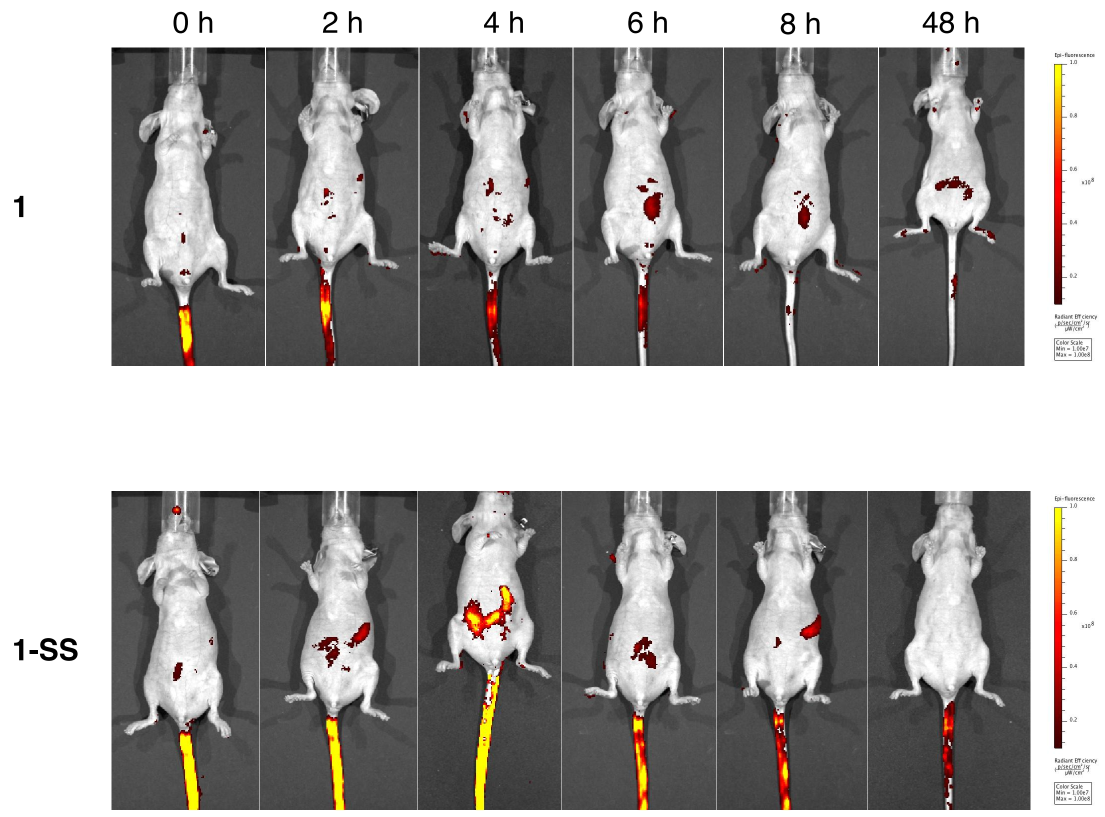

Figure S7: Additional time points comparing fluorescence between peptidic sensors **1** and **1–SS**.
